# The complete mitochondrial genome and phylogenetic analysis of *Illiberis pruni* Dyar, 1905 (Lepidoptera: Zygaenidae)

**DOI:** 10.1080/23802359.2022.2080598

**Published:** 2022-06-14

**Authors:** Yanbin Nan, Jingwen Peng, Kexing Cheng, Jiahui Guo, Zhiao Pei, Mingyan Ma, Yuantao Zhou

**Affiliations:** College of Agriculture and Animal Husbandry, Qinghai University, Xining, Qinghai, China

**Keywords:** *Illiberis pruni*, mitochondrial genome, sequence, phylogenetic analysis

## Abstract

*Illiberis pruni* is a leaf-eating pest that infests pear trees across all pear-producing regions of China. The present study, aimed to sequence the *I. pruni* mitochondrial genome (GenBank accession no. MZ726799) using the Illumina NovaSe Sequencing System to understand the population genetics, evolution, and taxonomy of *I. pruni* and other related species. The circular *I. pruni* mitochondrial genome was found to be 15,252 bps in length and comprised 38 sequence elements including 13 protein-coding genes (PCGs), 22 transfer RNA (tRNA) genes, 2 ribosomal RNA (rRNA) genes, and a putative control region (CR). Phylogenetic analysis revealed that *I. pruni* and *Illiberis ulmivora* are closely related, thereby indicating that their mitochondrial genes may share common ancestry.

*Illiberis pruni* classified under Zygaenidae, Lepidoptera, and commonly known as dumpling insect, is the primary leaf-eating pest of pear trees. The worm is endemic across all pear-producing regions of China, and mainly infests pear, peach, apple, and begonia trees among other fruit trees (Yan et al. 2020). The overwintering larvae feed on newly emerged flower and leaf buds (Li 2013). Consequently, the damaged flower buds mostly lose the ability to bloom, and retain brown wounds and holes. In summer, the newly hatched larvae feed on mesophyll. *I. pruni* infestations occur twice a year, the first round being more severe than the second. The former often results in drastic defoliation and fruit loss, thereby seriously impacting yield and tree potential. Scientists have proposed a number of preventive and control measures to combat damage caused by *I. pruni* infestations. For instance, natural enemies have been introduced in an attempt to suppress the population (Song 2014). Further, old bark is brushed, burned, or buried deep in autumn and winter to reduce resources available to overwintering insects (Wang 2017). The genetic structure and phylogenetic status of *I. pruni* have not been reported yet. This study, for the first time, reports the complete mitochondrial genome sequence of *I. pruni* and compares it with that of other insects to aid comprehension of the population genetics, evolution, and taxonomy of *I. pruni* and its related species.

The *I. pruni* specimens were collected in Xining, Qinghai Province, China (36°62 N, 101°77E), in May 2021. Samples was stored in the Insect Collection of the Entomology Lab, Qinghai University with specimen accession number ZYT-202105-03 (email: 1282186715@qq.com). Mitochondrial genomic DNA was extracted from an individual sample and genome sequencing was performed using the Illumina NovaSe Sequencing System (Illumina, San Diego, CA, USA). The sequencing read length was set at PE150. The mitochondrial genome was assembled using the SPAdes v3.10.1 software (http://cab.spbu.ru/software/spades/) (Bankevich et al. 2012), and gaps were filled with SSPACE 3.0 (Boetzer et al. 2011) and GapFiller 1.1 (Boetzer and Pirovano 2012). Post assemblage of the mitochondrial genome, annotation was achieved using MITOS (Bernt et al. 2013).

The complete *I. pruni* (GenBank accession number: MZ726799) mitochondrial genome was of typical structure and 15,252 bps in length. In the context of nucleotide composition, it comprised 39.94% adenosine (A), 42.32% thymine (T), 7.41% guanine (G), and 10.33% cytosine (C). The A:T ratio of 82.26% was found to be significantly higher than that of the G:C ratio of 17.74%. The AT-skews and GC-skews calculated for the major strands of the mitogenome were approximately −0.029 and −0.165, respectively. There were 38 sequence elements in total that comprised 13 protein-coding genes (PCGs), 22 transfer RNA (tRNA) genes, 2 ribosomal RNA (rRNA) genes, and a putative control region (CR). The 13 PCGs of *I. pruni* possessed the ATN (Met) start codon that is typical of invertebrate mitochondrial PCGs, except for cox1 that utilized a CGA initiation codon. Three of these genes (*nad1*, *nad2*, and *atp8*) used ATT, another four genes (*cox2*, *nad5*, *nad6*, and *cob*) used ATA, and the remaining five genes (*atp6*, *cox3*, *nad3*, *nad4*, and *nad4l*) had ATG. Of the 13 PCGs, 10 terminated with the typical TAN stop codon with nine genes (*nad1*, *nad2*, *atp6*, *atp8*, *cox3*, *nad3*, *cob*, *nad4l*, and *nad6*) using a TAA stop codon, while a single gene (*nad4)* used the TAT stop codon. Three genes (*cox1*, *cox2*, and *nad5*) ended with the incomplete codon T, which was completed by the addition 30 bp poly-A tail to the mRNA. All 22 tRNA genes that were scattered within the coding region, ranged from 63 to 74 bps in length, and could be folded into the typical clover secondary structure. The two rRNA genes of LrRNA and SrRNA genes were 1342 and 774 bps in length, respectively.

MEGA7 software was used to construct a phylogenetic tree and to analyze phylogenetic relationships between *I. pruni* and other insect species (Kumar et al. 2016). In addition, it was constructed using the maximum likelihood method, which was based on the 1,000 bootstraps, Jones-Taylor-Thornton model, uniform rates, partial deletion, nearest neighbor interchange heuristic method, and default automatic NJ/BioN. Figure 1 depicts the phylogenetic relationship between *I. pruni* and 19 other species. These included 4 members of Zygaenidae including *I. ulmivora*, *Eterusia aedea*, *Amesia sanguiflua,* and *Histia rhodope*; 3 species of Parnassiidae including *Parnassius nomion,* and *Parnassius bremeri*; 2 species of Gelechiidae including *Phthorimaea operculella* and *Pectinophora gossypiella*; 2 species of Nymphalidae including *Limenitis elwesi* and *Junonia almana*; 6 species of others Lepidoptera including *Cnaphalocrocis exigua* (Pyralidae), *Ampelophaga rubiginosa* (Sphingidae), *Omiodes indicata* (Crambidae), *Thyatira batis* (Thyatiridae), *Grapholita delineana* (Tortricidae), and *Euschemon rafflesia* (Euschemonidae). Two species of Diptera as outgroups, including *Haematopota vexativa* (Tabanidae) and *Pseudolimnophila brunneinota* (Limoniidae). All species were found to be clearly distinct from each other, with high bootstrap values, and the outgroups were grouped into small independent clades. We found that *I. pruni* and *I. ulmivora* formed a monophyletic clade with a bootstrapping rate of 100%, indicating that they are closely related, and that their mitochondrial genes may share a common ancestry. *Illiberis pruni* and *H. rhodope* belong to the same family, but the genomic sequence of *H. rhodope* has low similarity with that of *I. pruni*; thus, it is slightly distantly related to *I. pruni*. The results of this study will increase our understanding of, and drive further research in, insect population genetics.

**Figure 1.**
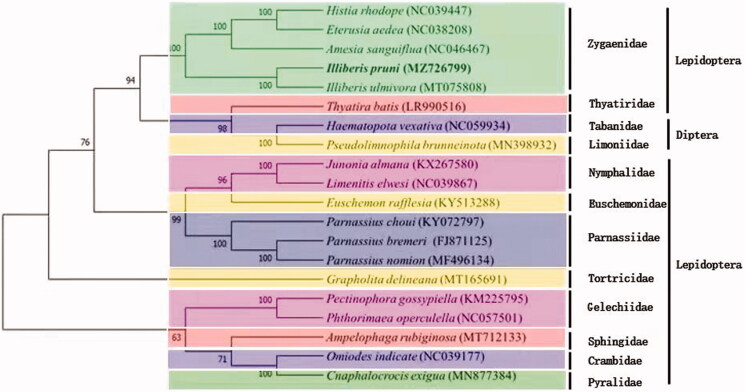
The phylogenetic tree inferred from 20 mitochondrial genomes. The number in the species name is the GenBank accession number. The *Illiberis pruni* complete mitochondrial genome obtained in this study is shown in bold. The number at each node is the bootstrap probability.

## Data Availability

The data that support the findings of this study can be found at the national center for biotechnology information GenBank publicly available on the web site, https://www.ncbi.nlm.nih.gov, reference number MZ726799. The associated BioProject, SRA, and Bio-Sample numbers are PRJNA757602, SRR15860541, and SAMN20968936, respectively.
